# Lifestyle, Cognitive, and Psychological Factors Associated With a Resilience Phenotype in Aging: A Multidimensional Approach on a Population-Based Sample of Oldest-Old (80+)

**DOI:** 10.1093/geronb/gbae132

**Published:** 2024-08-03

**Authors:** Elena Rolandi, Michele Rossi, Mauro Colombo, Laura Pettinato, Federica Del Signore, Virginia Aglieri, Gabriella Bottini, Antonio Guaita

**Affiliations:** Golgi Cenci Foundation, Abbiategrasso, Milano, Italy; Department of Brain and Behavioral Sciences, University of Pavia, Pavia, Italy; Golgi Cenci Foundation, Abbiategrasso, Milano, Italy; Golgi Cenci Foundation, Abbiategrasso, Milano, Italy; Golgi Cenci Foundation, Abbiategrasso, Milano, Italy; Golgi Cenci Foundation, Abbiategrasso, Milano, Italy; Fondazione Grigioni per il Morbo di Parkinson, Milano, Italy; Parkinson Institute Milan, ASST Gaetano Pini CTO, Milano, Italy; Department of Brain and Behavioral Sciences, University of Pavia, Pavia, Italy; Cognitive Neuropsychology Center Neuroscience Department, GOM Niguarda, Milano, Italy; Golgi Cenci Foundation, Abbiategrasso, Milano, Italy; (Psychological Sciences Section)

**Keywords:** Prevention, Cognitive reserve, Successful aging, Coping with illness/disability, Dementia

## Abstract

**Objectives:**

To investigate the determinants of resilience phenotype in aging, operationalized as the maintenance of cognitive, physical, and psychological health in very old individuals (80+), we investigated the structure and interrelated impact of the main resilience-enhancing factors, which are usually studied in separate research fields.

**Methods:**

Participants were older adults without dementia recruited for the fifth wave of the InveCe.Ab population-based cohort study (aged 83–87 years). Multidimensional evaluation comprised blood sampling, social and lifestyle survey, and geriatric and neuropsychological assessment. We classified resilient individuals as displaying normal cognition, functional independence, and mental health. First, we performed exploratory factor analysis (EFA) to examine the underlying structure of the relevant cognitive, lifestyle, physical, and psychological resilience-enhancing factors. The factors obtained were included as predictors of the resilience phenotype in the logistic regression model, controlling for sociodemographic and cumulative exposure to physical and psychosocial stressors, including COVID-19 infection.

**Results:**

Among the 404 enrolled participants, 153 (38%) exhibited the resilience phenotype. EFA resulted in the identification of six factors (59% of variance): cognitive reserve, affective reserve, insecure attachment, current lifestyle, physical reserve, and avoidant attachment. Among these factors, cognitive reserve, affective reserve, and current lifestyle significantly and independently predicted resilience status, controlling for cumulative exposure to age-related stressors and COVID-19 infection.

**Discussion:**

Our findings showed that, even in very old age, both early and late life modifiable factors affect individuals’ ability to adapt to the aging process, thus confirming the importance of a life-course approach to improve health outcomes in the aged population.

**Clinical Trials Registration Number:**

NCT01345110

## Introduction

The increase in life expectancy and the progressive aging of the general population are phenomena occurring worldwide (Christensen et al., 2009). Although cohort comparisons suggest that the debilitating effects of senescence are delayed by a decade, advanced adulthood continues to be associated with functional decline and health status problems, with important implications for individuals, society, and healthcare systems (Vaupel, 2010).

In this scenario, emerging preventive approaches highlighted the need to address the promotion of brain and body health, in contrast with the classical risk reduction model aimed at reducing disease incidence (Hachinski and Avan, 2022; Lisko et al., 2021). This paradigm shift is further supported by the fact that some of the most prevalent age-related diseases share the same risk factors, such as stroke, heart disease, dementia, and disability (Hachinski and Avan, 2022; Lisko et al., 2021). Thus, it is of great scientific interest and public health utility to investigate the determinants of positive health outcomes in very old individuals (over 80 years of age). Aging is a universal and deleterious process characterized by the progressive accumulation of biological changes that occur over time and which increase the probability of disease and death (Viña et al., 2007). Thus, maintaining health in very old age requires a continuous process of compensation and adaptation to low-intensity biological and psychosocial stressors.

Resilience is the ability to cope with and adapt to adverse events, diseases, and stressful situations. To assess resilience, at least two key factors must be considered: the presence of a stressor and a positive response/adaptation (Arenaza-Urquijo and Vemuri, 2018). The definition of stressor should be chosen depending on the context and goal of resilience research (Angevaare et al., 2020). A recent systematic review of the conceptual literature on resilience in aging research highlighted the presence of two chief perspectives: the most widely applied, defines resilience as a positive response to a highly intense stressor; whereas the most recent one describes it as the dynamic process of adapting and regaining equilibrium following low-intensity stressors over time (Angevaare et al., 2020). In line with this latter perspective, we conceptualized resilience during the aging process as the dynamic and multidimensional ability to counteract accumulating physical damage and psychosocial stressors, including COVID-19 infection, maintaining cognitive and mental health, and functional independence in later life. This definition of positive adaptation reflects a multidimensional conceptualization of health.

Conversely, previous studies investigating resilience in older adults have usually considered only one of these aspects (cognitive, psychological, or physical health). Broadly, we can identify three corresponding primary definitions of resilience in aging research (Abadir et al., 2023). The psychological resilience construct is derived from developmental psychology and is mostly studied as a protective factor against mental illness, such as depression (Laird et al., 2019). The American Psychological Association defines resilience as “the process and outcome of successfully adapting to difficult or challenging life experiences, especially through mental, emotional, and behavioral flexibility and adjustment to external and internal demands” (APA Dictionary of Psychology, n.d.). Cognitive resilience is a property of the brain that allows for sustained cognitive performance in the face of age-related changes and neurodegenerative diseases (Arenaza-Urquijo and Vemuri, 2018; Stern, 2012) and accounts for the discrepancy between observed and expected cognitive impairment associated with age or with a given degree of neuropathology (Arenaza-Urquijo and Vemuri, 2018; Stern, 2012). Finally, the construct of physical resilience, which has recently emerged in the biomedical literature, is the ability to recover or optimize function in the face of age-related losses or illnesses (Resnick et al., 2011; Whitson et al., 2016).

However, regardless of the outcome considered (cognitive, psychological, and physical), similar methodological limitations have been highlighted in each of the research areas. First, there is no consensus on the terminology and instruments used to assess resilience (Angevaare et al., 2020; Arenaza-Urquijo and Vemuri, 2018; Cosco et al., 2016). Most evidence comes from cross-sectional analyses of convenience samples. Finally, overlap and interrelations exist between the different predictors and proxies of resilience; thus, the study designs in the field must address the issues of low specificity, multidirectionality, and collinearity between measurements (Fratiglioni et al., 2020).

The present project addresses these limitations by jointly considering cognition, mental health, and functional autonomy as a single comprehensive multidimensional outcome, that we refer to here as the “resilience phenotype.” We were also able to concurrently assess the unique contributions of the main resilience-enhancing factors identified in previous studies. This notable aim was pursued by adopting a multidimensional approach on a population-based sample of the oldest-old (80+), an understudied but growing portion of the global population with increased vulnerabilities and specific care needs (Escourrou et al., 2020).

Specifically, we formulated two main research questions to achieve our aim:

RQ1. What is the underlying structure of the cognitive, psychological, and physical resilience-enhancing factors in the oldest-old without dementia?
RQ2. To what extent do the factors considered predict the resilience phenotype, controlling for proxies of cumulative exposure to physical and psychosocial stressors?

## Methods

### Study Design

**Figure 1.**
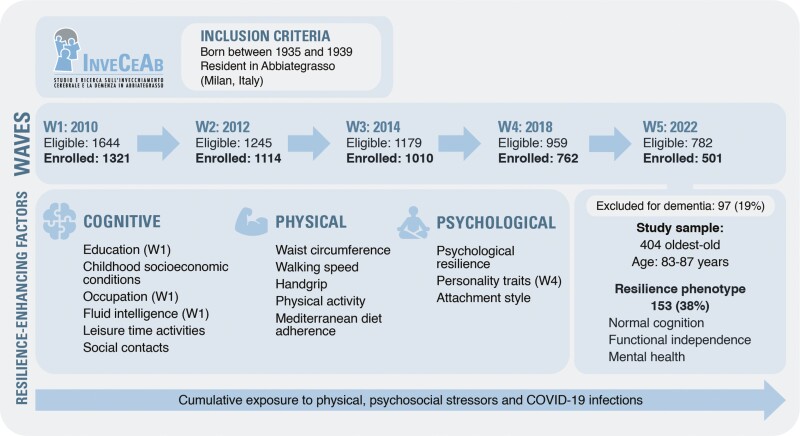
Flow diagram showing the InveCe.Ab study waves, participants selection, and measures of interest of the present study. In the upper part, we show the inclusion criteria for the population-based cohort study “InveCe.Ab.” In the middle, the flow chart of the participants in the five waves is shown. Below, there are the criteria used to define the resilience phenotype and the specific features of the study sample, selected among the participants to the fifth wave. In the resilience-enhancing factors box, we report the measures used as predictors of the resilience phenotype, grouped according to the corresponding domain (cognitive, physical, and psychological). Unless otherwise specified (in brackets), the measures were collected during the fifth wave (W5). In the bottom arrow, “Cumulative exposure to physical and psychosocial stressors” refers to the indices that combine the measures collected during the entire observation period (see “Stressors” subparagraph for details). InveCe.Ab = Invecchiamento Cerebrale in Abbiategrasso (i.e., Brain Aging in Abbiategrasso); COVID-19 = coronavirus disease 2019; W = wave.

This was a cross-sectional study on a population-based sample of older adults without dementia and aged between 83 and 87 years who participated in the fifth wave of the InveCe.Ab study, a population-based cohort enrolled in 2009 (Invecchiamento Cerebrale in Abbiategrasso, i.e., Brain Aging in Abbiategrasso; ClinicalTrials.gov, NCT01345110). Here, we mainly used data from the last assessment performed in 2022, which included all relevant variables for the resilience construct. However, in light of our aim, we reconstructed indices of cumulative exposure to stressors using data from the entire study period. Figure 1 shows the study design and measurements used for the present study, within the framework of the InveCe.Ab project.

### Participants and Procedures

Participants were recruited from the InveCe.Ab study, a population-based cohort of people born between 1935 and 1939 who lived in the city of Abbiategrasso when the study began (November 1, 2009). The aim was to estimate the incidence of dementia and to explore sociodemographic, clinical, and lifestyle factors associated with aging and dementia (Guaita et al., 2013). Participants enrolled underwent multidimensional assessments at baseline (*n* = 1,321), and after 2, 4, and 8 years; they engaged in blood sampling, geriatric visits, neuropsychological assessments, and social and lifestyle interviews.

For our purposes, all InveCe.Ab participants (age range: 83–87 years) who were alive and underwent at least one follow-up assessment after baseline were contacted again in 2022 to participate in the fifth wave of the study. When the study began (mid-February 2022), there were 782 eligible participants.

All the eligible individuals received a personalized letter explaining the study’s aims and procedures for the fifth wave. Then, trained personnel called each participant to provide further explanations and, in the case of acceptance, to schedule the appointments for a multidimensional assessment comprising blood sampling at home by a nurse, a social and lifestyle survey by phone lasting approximately 30 min, and in-person visits to the Golgi Cenci Foundation with the neuropsychologist and the physician (geriatrician or neurologist) on the same day, lasting a total of 3 hr. Visits could also be provided at home or in a nursing home to meet older adults’ needs.

The study procedures were carried out in accordance with the principles outlined in the Declaration of Helsinki of 1964 and the relevant amendments. The study was approved by the Ethics Committee of Milano Area 3 on January 19, 2022 (number: 26-19012022). Informed consent was signed before the study visits.

### Multidimensional Assessment

The geriatrician collected a guided and detailed medical history supported by clinical documentation, recorded current medications, reviewed the results of the blood tests, and performed a physical examination with special attention to neurologic signs and symptoms. Comorbidity was assessed with the Cumulative Illness Rating Scale (CIRS; Parmelee et al., 1995). Functional status was assessed with the Basic Activity of Daily Living (BADL) and Instrumental Activity of Daily Living (IADL) scales (Katz et al., 1970; Lawton and Brody, 1969).

Neuropsychologists administered the 15-item form of the Geriatric Depression Scale (GDS) and a comprehensive neuropsychological test battery to evaluate global cognition (Mini-Mental State Examination, MMSE) and the main cognitive domains: language (category fluency), memory (Rey-Auditory Verbal Learning Test), attention (attentional matrices), executive functions (Trail Making Test, Raven Coloured Progressive Matrices), visuospatial abilities, and constructional praxis (Clock Drawing Test; Rey-Osterrieth Complex Figure Test). Age- and education-adjusted scores were used according to Italian norms to determine the presence of impairment for each test. See the study protocol for more details on the instruments used (Guaita et al., 2013).

After the visits, the neuropsychologist and the physician made independent working diagnoses within the following categories: dementia, based on DSM-V criteria (Sachdev et al., 2014); mild cognitive impairment, based on the Petersen criteria (Petersen, 2004); cognitive impairment—not dementia (Chertkow et al., 2008); clinically relevant depression or subthreshold depression (Vaccaro et al., 2017); and psychosis (ICD10: F20–F29). Participants without cognitive impairment and without major mental disorders (clinically relevant depression or psychosis) were classiﬁed as cognitively normal.

After multidimensional assessment completion, an expert geriatrician (A.G.) together with a clinical neuropsychologist (L.P.) reviewed all individual records, and a final diagnosis within the aforementioned categories was reached.

### Resilience Phenotype

For the present study, we included all participants without dementia based on the multidimensional assessment performed. Then, we classified resilient individuals as those who maintained normal cognition, mental health, and functional independence in very old age (83–87 years). This represents a positive adaptation to the aging process, considering that all of the participants are oldest-old in a specific age quintile according to the study design. Normal cognition was ascertained by the agreement of the geriatrician and the neuropsychologist who reviewed the individual charts, as specified in the subparagraph on multidimensional assessment. Functional independence was determined if all the BADLs were preserved. Mental health was ascertained by the absence of clinically relevant depression or psychosis at the final diagnosis.

### Resilience-Enhancing Factors

During the fifth wave, to enrich the characterization of resilience in aging, we added specific scales to the standard InveCe.Ab instruments are detailed in the study protocol (Guaita et al., 2013).

Table 1 displays the measures used as predictors of resilience in the present study. Unless otherwise specified, we used the data collected during the fifth wave (2022). We grouped the measures into the three chief dimensions of cognitive, physical, and psychological health, based on the a priori hypothesis about their potential actions in relation to the different dimensions. In the cognitive domain, we included variables classically used as indirect proxies of cognitive reserve, representing the amount and complexity of cognitive-stimulating activity performed throughout one’s life, such as years of education, occupational level, leisure time cognitive activities, and social contact later in life (Pinto et al., 2024). As a measurement of early-life cognitive enrichment, we used the childhood socioeconomic condition indicator (Aartsen et al., 2019). Moreover, because the intelligence quotient is frequently employed as a cognitive reserve proxy, we used the raw baseline score of the Raven Coloured Progressive Matrices (Basso et al., 1987), which assesses logical reasoning and is considered a measure of fluid intelligence (one of the two components of general intelligence). In the physical health domain, we included self-reported measures of physical activity and Mediterranean diet adherence (Schröder et al., 2011) due to their established protective effects against several aging-related diseases. We also included waist circumference, handgrip test (Lemmink et al., 2001), and walking speed test (Camicioli et al., 1997), in line with recent contributions that suggest measuring physical functionality directly through standardized performance and strength tests, to better capture the real biology of the function (Patrizio et al., 2021).

**Table 1.** Description of the Measures Used to Predict Resilience

Measure of interest	Description	Score
**Cognitive health**		
Education	Number of years of formal education completed.	Continuous number
Childhood socioeconomic conditions (Aartsen et al., 2019)	Socioeconomic conditions at the age of 10, based on 4 binary indicators (advantaged/disadvantaged). Disadvantaged conditions were: (i) low occupational position of the main breadwinner; (ii) less than 10 books at home; (iii) household overcrowding; (iv) low housing quality.	Sum of the 4 indicators ranging from most disadvantaged (0) to most advantaged (4).
Occupation	Main occupation during the life course (collected at baseline, W1), classified into 4 levels corresponding to increase cognitive complexity (housekeeper; workman, farmer; office worker, sales worker; nurse; professional, teacher, manager).	Categorical variable ranging from 1 to 4.
Fluid intelligence (Basso et al., 1987)	The Raven Coloured Progressive Matrices test score at baseline (W1), assessing logical reasoning, one of the 2 components of general intelligence. Participants have to choose among 6 alternatives, which one best fits into an incomplete pattern.	Raw test score, range: 0–36.
Leisure time activities	Self-report questionnaire on leisure time activities performed in the past year, composed of 15 items (e.g.: voluntary work, going to the theatre, reading books).	Sum of the responses. Responses are on a Likert scale ranging from 0 to 2 (no, yes sometimes, yes often/regularly).
Social contact	Self-report questionnaire on the monthly frequency of in-person meetings with no cohabiting persons grouped according to prespecified categories: neighbors, brothers, sisters, daughters, sons, friends, other.	Sum of the responses. Responses are on a Likert scale ranging from 0 to 3 (not present or cohabiting, never or less than once a month, 1–3 times per month, 4 and more times per month).
**Physical health**		
Waist circumference	Measured at the end of normal expiration at the midway between the lower rib and the iliac crest	Continuous number in centimeters
Walking speed (Camicioli et al., 1997)	Participants were asked to rise from a chair and to walk back and forth along an indicated distance of 5 m with no pausing, and then to sit down again.	Seconds needed to complete the task
Handgrip (Lemmink et al., 2001)	The standard “Groningen Elderly Test” was followed to measure hand and forearm muscles’ maximal isometric strength.	The best of the 3 performances expressed in kilograms.
Physical activity	Self-report questionnaire on the weekly frequencies of physical activity performed in the past month composed of 9 items (e.g.: running, cycling, swimming).	Sum of the responses. Responses are on a Likert scale ranging from 0 to 3 (never, once a week, twice a week, 3 or more times per week).
Mediterranean diet adherence (Schröder et al., 2011)	The Mediterranean Diet Adherence Screener (MEDAS) score, assessing on binary responses to the daily consumption of: olive oil, vegetables, fruits, red meat, butter/margarine/cream, sugary drinks; and weekly consumption of wine, pulses, fish/seafood, commercial pastries, nuts, and use of tomato sauces/garlic/onion/leeks sautéed in olive oil.	Sum of the responses to the 14 items.
**Psychological health**		
Psychological resilience (Callegari et al., 2016)	The 14-item resilience scale (RS-14) total score. It investigates five characteristics of the resilience core: purpose (e.g.: “My life has meaning”), perseverance (e.g.: “I am determined”), self-reliance (e.g.: “I usually manage one way or another”), equanimity (e.g.: “I usually take things in stride”), and authenticity (e.g.: “I am friends with myself”).	Sum of the responses. Possible responses are on a Likert scale ranging from 1 (strongly disagree) to 7 (strongly agree).
Personality traits (Dazzi et al., 2004)	The short form of the Eysenck Personality Questionnaire–Revised (EPQ-SF-R), administered during the fourth wave (W4). The scale is composed of 4 subscales to measure three personality traits (extraversion, neuroticism, psychoticism) and a control scale, which measures social desirability (Lie).	Raw score of the three personality traits subscales. Each subscale is composed of 12 items with a binary response (yes/no).
Attachment style (ASQ) (Fossati et al., 2003)	The Attachment Style Questionnaire (ASQ), composed of 40 items measuring the five dimensions of adult attachment: Confidence (8 items; e.g.: “I feel confident about relating to others”), Discomfort with Closeness (10 items; e.g.: “I find it hard to trust other people”), Need for Approval (7 items; e.g.: “It’s important to me that others like me”), Preoccupation with Relationships (8 items; “Other people often disappoint me”), Relationships as Secondary (7 items; “My relationships with others are generally superficial”). High scores on the Confidence scale reflect a secure attachment style. Higher scores on the other subscales denote an insecure attachment style (avoidant/dismissing and anxious/preoccupied).	Score of the five ASQ subscales (sum of the responses for each scale). Responses are on a Likert scale ranging from 1 (totally disagree) to 6 (totally agree).

Finally, as predictors of psychological health, we chose measurements known to increase individuals’ ability to adapt to adverse and stressful situations and protect against mental illness. The 14-item Resilience Scale measures self-reported adaptive coping styles to counteract adversity, with specific questions on five dimensions (Callegari et al., 2016): purpose, perseverance, self-reliance, equanimity, and existential aloneness (authenticity). These are all psychosocial characteristics associated with high resilience among older adults (Laird et al., 2019; MacLeod et al., 2016). Personality traits and attachment styles are stable individual traits shaped by gene‒environment interactions in early life and are associated with the risk of mental illness during one’s life (Laird et al., 2019). We measured three personality traits using the Eysenck Personality Questionnaire–Revised (EPQ-SF-R; Dazzi et al., 2004): extraversion (a tendency to be sociable, carefree, convivial, easy-going, and impulsive), neuroticism (low emotional stability and a predisposition toward experiencing negative affect), and psychoticism (a tendency to be hostile, untrusting, unemotional, lacking in empathy, and unfriendly). Adult attachment styles refer to an individual’s emotional regulation abilities, which arise from the intimate bonds built with caregivers very early during infancy, modulating the internal state and subsequent behaviors in close relationships (Bartholomew and Horowitz, 1991). An early supportive and responsive social environment, leading to a secure attachment profile, encourages both mental and cognitive health (Walsh et al., 2019). Conversely, insecure attachment has been linked to dysregulated physiological responses to stress, risky health-related behaviors, and susceptibility to serious physical illness (Pietromonaco and Beck, 2019).

### Stressors

We used indices based on instruments administered in each wave to gauge the cumulative exposure to physical and psychosocial stressors during the aging process. We also include COVID-19 infection as an important source of stress, especially for the oldest individuals, who are at increased risk of hospitalization and death due to COVID-19 (Minnai et al., 2022).

Physical stressors were based on two comorbidity measures: the CIRS severity index and the number of drugs used. The CIRS (Parmelee et al., 1995) is a standardized tool compiled by a physician to rate the level of pathology and impairment of 13 major organ groups and in the psychiatric/behavioral domain on a scale ranging from 1 (no impairment) to 5 (extremely severe impairment). The severity of overall physical illness was computed as the mean of the 13 items (excluding the psychiatric/behavioral domain). For our purposes, we used the average CIRS severity score of all available assessments for each participant.

Moreover, during the geriatric visit, the number of pharmacologically active ingredients consumed in the past 2 weeks was recorded, and a list was compiled of 53 prespecified principles chosen among those drug categories most likely to interfere with cerebral activity. We used the average number of pharmacologically active ingredients recorded in all available assessments.

Psychosocial stressors were represented by the score of the Geriatric Adverse Life Events Scale (GALES) which investigates the stressful life events experienced over the previous 12 months with a 26-item checklist (Devanand et al., 2002). For the present study, we measured cumulative exposure to psychosocial stressors as the sum of the adverse events reported in all available assessments.

Finally, as a potential stressor, we included contracting COVID-19, which was assessed as a yes/no question during the geriatric visit in 2022.

### Statistical Analysis

All statistical analyses were performed using SPSS Statistics 20.0 (IBM SPSS Statistics for Windows, version 20.0, BM Corp. Released 2011, Armonk, NY, US). Preliminarily, descriptive univariate analysis was performed to compare the resilient and non-resilient subgroups using Student’s *t*-test for continuous variables and the chi-squared test for categorical ones.

To address our research questions, we performed several steps of analysis. First, we were interested in exploring the underlying structure of the measures considered to be predictors of resilience (see Table 1 for details). To make the statistical analyses more robust and to include the entire sample evaluated, we handled missing data by performing a data imputation through Predictive Mean Matching with Full Conditional Specification (MCMC), which specifies a predictive model for each missing variable considering all other relevant variables to be predictors (i.e., the six factors, sociodemographics, stressors, and the main clinical scales of MMSE, GDS, BADL). This ensures a plausible posterior predictive distribution. Then, we performed an exploratory factor analysis (EFA) because, to the best of our knowledge, no previous study has concurrently assessed all these physical, cognitive, and psychological factors in the same sample of oldest-old individuals. We examined the correlation matrix for randomness using Bartlett’s test of sphericity, and the KMO statistic had to be greater than or equal to 0.50. The correlation matrix was factored using the principal axis factoring method after we determined that it could be factored. We assumed that the factors considered would be correlated, so an oblimin rotation was used. We applied Kaiser’s criterion (an eigenvalue greater than 1) to establish how many factors should be retained. We defined a threshold of 0.3 for variable loadings to justify the inclusion of the factors obtained and eventually rerun the analysis accordingly (Osborne, 2014). To analyze the factors as new variables, we extracted the scores using Bartlett’s method.

This step of analysis provided answers to RQ1 and reduced the dimensions of the dataset by obtaining a limited number of factors with higher internal consistency and low intercorrelation, thus addressing the issue of multicollinearity among the predictors.

Second, to investigate the relative importance of each factor in explaining resilience (RQ2), we constructed a logistic regression model with the resilience phenotype (1 = yes; 0 = no) as the dependent variable and factor scores as the independent variables, with sociodemographics (age, sex) and stressor indices as covariates. To test the performance of the models, we studied sensitivity and specificity using ROC curves.

## Results

### Study Sample

Recruitment took place between mid-February 2022 and January 2023. Among the 782 eligible individuals, 27 died (3.5%), 52 were unreachable (6.6%), and 202 refused to take part (25.8%). Thus, 501 participants (64.1% of the eligible population) participated in the fifth wave, and 97 received a final diagnosis of dementia (19.4%), resulting in a sample of 404 oldest-old individuals without dementia who were eligible for the present investigation. Among them, 153 were categorized as resilient (38%) based on prespecified criteria. Before imputation, 11% of the values in the dataset were missing. Table 2 shows the comparisons of the measures of interest according to resilience status. Resilient individuals were more likely to be male and slightly younger. Overall, they showed a more favorable profile for most of the measures used as predictors of resilience. They were relatively healthier based on the CIRS index and on the number of drugs assumed, whereas no differences were found in terms of exposure to stressful life events or COVID-19 infection.

**Table 2.** Participant Characteristics and Comparisons of the Measures of Interest According to the Resilience Phenotype Status

Variables	Controls	Resilient	Total	Test	*p* ^*^
	*n* = 251	*n* = 153	*N* = 404		
**Sociodemographics**					
Age	84.4 (1.3)	84.1 (1.4)	84.3 (1.3)	2.036	**.043**
Sex (Female)	170 (68%)	77 (50%)	247 (61%)	11.395	**.001**
**Resilience-related factors**					
Education level	6.9 (3.2)	8 (3.4)	7.3 (3.3)	−3.212	**.001**
Childhood socioeconomic conditions	1.0 (1.0)	1.4 (1.1)	1.2 (1.1)	−3.066	**.002**
Occupation	2.3 (0.9)	2.5 (0.8)	2.4 (0.9)	−3.276	**.001**
Fluid intelligence	27 (4.8)	29.1 (4.5)	27.8 (4.8)	−4.451	**.000**
Leisure time activities	4.9 (2.7)	6.3 (3.0)	5.4 (2.9)	−4.801	**.000**
Social contact	8.5 (3.9)	9.5 (3.8)	8.9 (3.9)	−2.616	**.009**
Waist circumference	96.9 (13.0)	96.3 (12.1)	96.7 (12.6)	0.435	.664
Walking speed	20.8 (12.2)	15.5 (4.0)	18.8 (10.2)	6.259	**.000**
Handgrip	22.1 (6.4)	25.1 (7.1)	23.3 (6.8)	−4.155	**.000**
Physical activity	2.4 (2.9)	3.8 (2.9)	2.9 (3.0)	−4.686	**.000**
Mediterranean diet adherence	5.9 (2.2)	6.5 (2.1)	6.1 (2.2)	−2.840	**.005**
Psychological resilience	77.2 (14.3)	83.6 (11.2)	79.6 (13.6)	−5.043	**.000**
Extraversion	6.7 (2.6)	7.1 (2.5)	6.8 (2.6)	−1.635	.103
Neuroticism	4.2 (3.0)	3.3 (2.7)	3.8 (2.9)	2.992	**.003**
Psychoticism	2.2 (1.4)	2.3 (1.4)	2.2 (1.4)	−0.303	.762
Confidence	34.7 (4.1)	35.4 (4.0)	34.9 (4.1)	−1.588	.113
Discomfort with closeness	40.8 (5.9)	40.1 (6.2)	40.6 (6.1)	1.159	.248
Need for approval	24.2 (5.2)	22.5 (5.2)	23.6 (5.3)	3.341	**.001**
Preoccupation with relationships	30 (5.4)	28.7 (5.5)	29.5 (5.5)	2.321	**.021**
Relationships as secondary	23 (6.1)	23.5 (6.3)	23.2 (6.2)	−0.810	.418
**Stressors**					
CIRS severity score	1.6 (0.2)	1.5 (0.2)	1.6 (0.2)	4.186	**.000**
GALES	10.2 (4.2)	9.7 (4.3)	10 (4.2)	0.886	.377
Pharmacological active ingredients	4.5 (2.3)	3.5 (2.0)		4.394	**.000**
COVID-19 exposure	56 (23%)	38 (25%)	94 (24%)	0.113	.737

*Notes:* CIRS = Cumulative Illness Rating Scale; COVID-19 = coronavirus disease 2019; GALES = Geriatric Adverse Life Events Scale. The means (standard deviations) for continuous variables and the frequencies (percentages) for categorical variables are reported.

^*^Statistical significance according to the Welch test for continuous variables and the chi-square test for categorical variables. Significant differences are reported in bold.

### The Structure of the Resilience-Related Factors

The first EFA on the 20 selected variables resulted in six factors, with two variables showing loadings of less than 0.3 (neuroticism and psychoticism). We reran the EFA excluding these variables, confirming the number and composition of factors resulting from the first analysis.

The correlation matrix was not random according to the results of Bartlett’s test of sphericity: χ^2^(190) = 1,322.818, *p* < .001; in addition, the KMO statistic was 0.672, above the minimal threshold for factor analysis. As a result, we decided that factor analysis would be appropriate for the correlation matrix.

Kaiser’s criterion suggests retaining six factors that, after rotation, accounted for 59% of the total variance. Table 3 shows the factor loadings from the pattern matrix. Factor 1 (17% of the total variance) included all the variables related to cognitive enrichment and stimulation in early and middle life, namely, education, occupation, childhood socioeconomic conditions, and fluid intelligence; thus, it was named “cognitive reserve.” Factor 2 (12% of the total variance) grouped psychological resilience, confidence, and extraversion; thus, we named it “affective reserve.” Factor 3 (9% of the total variance) included the need for approval, relationship as secondary, and preoccupation with relationships subscales of the Attachment Style Questionnaire (ASQ); this factor was named “insecure attachment.” Factor 4 (8% of the total variance) was named “current lifestyle” because it included physical activity, leisure time activities, social contact, adherence to the Mediterranean diet, and walking speed. Considering the direction of the loadings, we changed the sign of this factor to improve the interpretative reading of its relationship with the outcome (i.e., higher scores represent a more active lifestyle). Factor 5 (7% of the total variance), including handgrip strength and waist circumference, can be defined as “physical reserve.” Finally, Factor 6 (6% of the total variance) included only the ASQ subscale of discomfort with closeness; thus, it can be considered a measure of “Avoidant Attachment.” The correlation among the factors after rotation ranged between −0.216 and 0.183 (see Supplementary Table S1).

**Table 3.** Results of Exploratory Factor Analysis Showing Variable Loadings of the Resilience-Related Factors Considered After Selection

Variables	Factor loading					
	1	2	3	4	5	6
Education	**0.942**	0.023	0.006	0.040	−0.011	−0.069
Occupation	**0.608**	0.019	0.078	0.041	0.074	−0.097
Childhood socioeconomic conditions	**0.470**	0.056	−0.002	−0.010	−0.055	0.064
Fluid intelligence	**0.375**	−0.085	−0.173	−0.110	0.034	−0.004
Psychological resilience	−0.025	**0.734**	−0.073	−0.189	0.071	0.187
Confidence	0.038	**0.660**	−0.003	0.107	−0.051	−0.086
Extraversion	0.038	**0.404**	0.102	−0.068	0.087	−0.056
Need for approval	−0.051	−0.071	**0.845**	−0.038	−0.128	−0.029
Relationships as secondary	−0.049	0.121	**0.625**	−0.053	0.179	0.124
Preoccupation with relationships	0.061	0.022	**0.511**	0.094	−0.100	0.069
Physical activity	−0.044	0.035	0.098	**−0.664**	−0.043	−0.101
Leisure time activities	0.153	0.028	−0.130	**−0.535**	−0.020	0.110
Social contact	−0.172	0.151	−0.019	**−0.439**	0.045	−0.096
Mediterranean diet adherence	0.063	−0.105	0.019	**−0.349**	0.130	−0.100
Walking speed	−0.095	−0.041	0.106	**0.383**	0.097	−0.151
Handgrip	0.119	−0.003	0.116	−0.226	**0.536**	−0.115
Waist circumference	−0.044	0.071	−0.131	0.133	**0.561**	0.073
Discomfort with closeness	−0.113	−0.076	0.243	0.065	0.041	**0.667**

*Notes*: Loadings above the threshold set for inclusion in the factors are reported in bold (absolute value of 0.300 or higher).

### Predictors of Resilience Phenotype

Table 4 displays the results of the logistic regression model. Cognitive reserve, affective reserve, and current lifestyle are the factors that significantly and independently predict the resilience phenotype (*p* < .05), controlling for sociodemographics and cumulative exposure to physical stressors, stressful life events during the aging process, and COVID-19 infection, with an AUC of 0.70 (95% CI: 0.64–0.76).

**Table 4.** Results of the Logistic Regression Model to Test the Prediction of Resilience Phenotype

Variable	*B*	SE	Wald	*p*	OR	95% CI	
						Lower	Upper
Age	0.000	0.095	0.000	.999	1.000	0.831	1.204
Sex	−0.091	0.363	0.063	.802	0.913	0.448	1.859
Cognitive reserve	0.360	0.128	7.888	**.005**	1.434	1.115	1.844
Affective reserve	0.294	0.118	6.207	**.013**	1.342	1.065	1.690
Insecure attachment	−0.166	0.118	1.993	.158	0.847	0.673	1.067
Current lifestyle	0.511	0.124	16.929	**.000**	1.667	1.307	2.126
Physical reserve	0.123	0.124	0.981	.322	1.130	0.887	1.441
Avoidant attachment	0.127	0.102	1.547	.214	1.136	0.929	1.387
CIRS severity score	−0.103	1.136	0.008	.927	0.902	0.097	8.357
Pharmacological active ingredients	−0.176	0.100	3.080	.079	0.838	0.689	1.021
GALES	0.013	0.031	0.168	.682	1.013	0.953	1.077
COVID-19 exposure	−0.161	0.298	0.290	.590	0.852	0.475	1.528

*Notes*: CI = confidence interval; CIRS = Cumulative Illness Rating Scale; COVID-19 = coronavirus disease 2019; GALES = Geriatric Adverse Life Events Scale; OR = odds ratio; SE = standard error.

## Discussion

The present study aimed to explore the structure and impact of the predictors of a resilience phenotype in aging, conceptualized as the multidimensional and dynamic ability to counteract the accumulating damage and stressors that characterize the aging process, maintaining normal cognition, psychological health, and functional independence in very old age.

Among the participants, 38% were resilient. Compared with non-resilient individuals, resilient ones are not spared from stressful life events, including COVID-19 infection. The independent predictors of the resilience phenotype were cognitive reserve, affective reserve, and current lifestyle, controlling for cumulative exposure to physical and psychosocial stressors across a 12-year period.

### Features of Resilient Participants

The “resilience phenotype” was directly ascertained through a comprehensive multidimensional assessment in 38% of the sample under study, which was composed of individuals aged between 82 and 87 years who were without dementia. Resilient individuals were more likely to be male and slightly younger, despite the narrow age range considered. As expected, and in line with our hypothesis, they showed a more favorable profile in most of the measures considered to be predictors of resilience. Moreover, they were slightly healthier based on comorbidity measures. At the same time, they did not differ from the non-resilient subgroup for exposure to stressful life events during the observation period (12 years) or for COVID-19 infection.

### The Structure of the Resilience-Related Factors

The first finding of the study pertains to the structure of the main cognitive, psychological, and physical resilience-enhancing factors in old age (RQ1). For the exploratory factor analysis, we grouped the selected variables into six separate factors in a predictable and plausible way, favoring a straightforward interpretation of the underlying constructs. The cognitive reserve factor is composed of variables related to early-life cognitive enrichment, with education showing the highest loading, in line with previous research (Pinto et al., 2024). The affective reserve factor contains both trait-like factors shaped by gene—environment interactions in early life (attachment and personality traits) and state-like factors (i.e., belief and coping measured by the RS-14) known to protect against depression later in life (Laird et al., 2019). All self-report questionnaires on current habits and activities load primarily on the same factor, together with the walking speed test, a key indicator of physical health in older adults strictly related to neurosensory, muscle-skeletal, and cardiometabolic functions (Mount et al., 2019). Hence, this could be viewed as a comprehensive measure of adherence to a healthy lifestyle later in life, an established protective factor against dementia and other age-related diseases (Kivipelto et al., 2018). Finally, the factor “physical reserve” contains two measures exclusively related to physical health and functionality, namely, waist circumference and handgrip strength.

The loading values indicate direct relationships of both waist circumference and strength with the underlying factor, implying that a common proxy for overweight could be protective in our sample. This finding is in line with prior studies reporting an age-dependent relationship between body mass index and dementia risk over the course of life (Fratiglioni et al., 2020; Livingston et al., 2020; Rolandi et al., 2020).

Taken together, these findings contribute to advancing theoretical knowledge on the structure of physical, lifestyle, cognitive, and psychosocial protective factors in old age. Indeed, to the best of our knowledge, no previous study has concurrently measured all these independent but interrelated resilience-enhancing factors in the same sample of older adults. From a methodological standpoint, this first step of the analysis was crucial to reduce the dimensionality of our dataset to obtain a limited number of relevant predictors for the resilience phenotype, with high internal consistency and low intercorrelation, thus addressing the issue of multicollinearity among the measures of interest (Fratiglioni et al., 2020).

### Predicting the Resilience Phenotype

Cognitive reserve, affective reserve, and current lifestyle significantly and independently predicted the resilience phenotype in our sample of oldest-old people, with similar impacts on the probability of being resilient and controlling for cumulative exposure to physical and psychosocial stressors during the study period, from 70–75 years to 83–87 years of age (see Figure 1). Notably, we derived these findings from multivariable models of primary data, allowing us to directly control for the main confounding factors and to estimate the unique contribution of each predictor.

Cognitive reserve and modifiable lifestyles were established as protective factors against dementia; several theoretical and empirical models have highlighted their interrelationship in determining better cognitive outcomes during the aging process (Arenaza-Urquijo et al., 2015; Song et al., 2022).

The original finding of the present study is that the affective reserve factor—composed of variables typically related to mental health and psychological wellbeing—exerts a comparable and independent protective effect in determining resilient aging. A recent review focused on the protective effect of secure attachment on dementia introduced the concept of affective reserve to explain how favorable affective resources might protect against cognitive decline (Walsh et al., 2019). Our results could provide the first experimental insight into the existence of such protective factor in the context of aging.

Our results also have crucial translational implications. First, they further support the importance of being ambitious about disease prevention and promotion of successful aging, in line with the life-course approach for reducing dementia risk (Fratiglioni et al., 2020). Indeed, both the cognitive and affective reserve factors identified here are mostly composed of measures related to early-life exposure, which may be targeted by specific public health interventions to encourage an enriched, safe environment for younger generations. In this view, a new preventive model termed “young adult brain capital” focused on the importance of optimizing young adults’ brains and mental health to empower the next generation to become active agents in reducing their own risk of developing age-related diseases (Farina et al., 2023).

At the same time, we further confirm that it is never too late to start prevention by showing that the maintenance of an active, healthy lifestyle in very old age (80+) is an independent contributor to the resilience phenotype. This age group is a fast-growing portion of the population, posing an enormous challenge for health and social care systems but receiving limited attention in research studies. In line with our findings, a recent systematic review—which included 27 observational cohort studies—showed that eating a healthy diet with plenty of fruits and vegetables, engaging in physical and leisure/social activities, or having a combination of these lifestyle habits is associated with improved cognitive health in the oldest-old individuals (Ye et al., 2023). Targeting lifestyle habits to improve health and wellbeing (rather than to reduce disease incidence) throughout the life course may be the most effective and parsimonious approach, with positive outcomes for a broad range of age-related diseases (Hachinski and Avan, 2022).

### Strengths and Limitations

The present study has several methodological strengths. To the best of our knowledge, this is the first attempt to concurrently assess the structure and impact of the chief resilience-enhancing factors in the same cohort, thus allowing us to measure their direct and independent effects on the outcome of interest. Another original aspect is the operationalization of the resilient outcome as a multidimensional construct encompassing physical, cognitive, and psychological health. This is due to the opportunity to rely on a population-based cohort prospectively followed with comprehensive geriatric and neuropsychological assessments. At the same time, we bridge theoretical knowledge from different but interrelated research fields, offering practical suggestions to prevent age-related diseases and disabilities. Finally, we were able to recruit and evaluate hundreds of individuals aged between 83 and 87 years through a personalized approach and reciprocal trust consolidated over 12 years (Guaita et al., 2013). As such, we have provided important information on a selected portion of the aged population, which has displayed the greatest growth in recent years due to increased life expectancy but is rarely or marginally included in studies on aging (Ye et al., 2023).

Some limitations should also be discussed. First, although relying on a longitudinal cohort, for our aim, we were able to perform only cross-sectional analysis because we included some measures of interest only in the most recent waves of assessment (2018: personality traits, adherence to the Mediterranean diet; 2022: childhood socioeconomic conditions, handgrip strength, psychological resilience, and attachment style). Moreover, we focused on a narrow age range in a specific geographic area, thus reducing the generalizability of our findings. Finally, the current study adopted a new definition of resilience, thus it is important to replicate the results in other national contexts/samples whereas using a similar operationalization.

## Conclusions and Future Directions

The present project enriched the understanding of how lifestyle, cognitive, and psychological factors independently affect individuals’ ability to adapt to the aging process by maintaining physical, cognitive, and mental health. We confirmed that cognitive reserve and current lifestyle are vital drivers of resilient outcomes in a population-based sample of oldest-old individuals. We further found that a new emerging construct, termed affective reserve, exerts a comparable protective effect. These findings, as previously discussed, have direct practical implications for the prevention of dementia and age-related diseases. Future studies on longitudinal data and in different contexts are needed to further confirm these findings because facing the challenge of an aged population is a global issue. Moreover, the present study, by revealing the shared and specific variance of a wide array of resilience-enhancing factors, may inform future research on the biological mechanisms underlying the protective effects of these factors in aging. Finally, this approach to investigating different factors pertinent to graceful or pathological aging has inspired the development of a cognitive and biological model from the perspective of a historical lifespan.

## Supplementary Material

Supplementary data are available at *The Journals of Gerontology, Series B: Psychological Sciences and Social Sciences* online.

gbae132_suppl_Supplementary_Materials

## Data Availability

The datasets used and analyzed during the current study are available from the corresponding author upon reasonable request. The Inve.Ce.Ab study was preregistered on ClinicalTrials.gov (https://clinicaltrials.gov/study/NCT01345110).

## References

[CIT0001] Aartsen, M. J., Cheval, B., Sieber, S., Van der Linden, B. W., Gabriel, R., Courvoisier, D. S., Guessous, I., Burton-Jeangros, C., Blane, D., Ihle, A., Kliegel, M., & Cullati, S. (2019). Advantaged socioeconomic conditions in childhood are associated with higher cognitive functioning but stronger cognitive decline in older age. Proceedings of the National Academy of Sciences of the United States of America, 116(12), 5478–5486. 10.1073/pnas.1807679116 30804194 PMC6431198

[CIT0002] Abadir, P. M., Bandeen-Roche, K., Bergeman, C., Bennett, D., Davis, D., Kind, A., LeBrasseur, N., Stern, Y., Varadhan, R., & Whitson, H. E. (2023). An overview of the resilience world: Proceedings of the American geriatrics society and national institute on aging state of resilience science conference. Journal of the American Geriatrics Society, 71(8), 2381–2392. 10.1111/jgs.18388 37079440 PMC10523918

[CIT0003] Angevaare, M. J., Roberts, J., van Hout, H. P. J., Joling, K. J., Smalbrugge, M., Schoonmade, L. J., Windle, G., & Hertogh, C. M. P. M. (2020). Resilience in older persons: A systematic review of the conceptual literature. Ageing Research Reviews, 63, 101144. 10.1016/j.arr.2020.101144 32835890

[CIT0004] APA Dictionary of Psychology. (n.d.). Retrieved January 18, 2024, from https://dictionary.apa.org/resilience

[CIT0005] Arenaza-Urquijo, E. M., & Vemuri, P. (2018). Resistance vs resilience to Alzheimer disease. Neurology, 90(15), 695–703. 10.1212/WNL.0000000000005303 29592885 PMC5894932

[CIT0006] Arenaza-Urquijo, E. M., Wirth, M., & Chételat, G. (2015). Cognitive reserve and lifestyle: Moving towards preclinical Alzheimer’s disease. Frontiers in Aging Neuroscience, 7(August), 134. 10.3389/fnagi.2015.00134 26321944 PMC4530312

[CIT0007] Bartholomew, K., & Horowitz, L. M. (1991). Attachment styles among young adults: A test of a four-category model. Journal of Personality and Social Psychology, 61(2), 226–244. 10.1037//0022-3514.61.2.226 1920064

[CIT0008] Basso, A., Capitani, E., & Laiacona, M. (1987). Raven’s coloured progressive matrices: Normative values on 305 adult normal controls. Functional Neurology, 2(2), 189–194. 3666548

[CIT0009] Callegari, C., Bertù, L., Lucano, M., Ielmini, M., Braggio, E., & Vender, S. (2016). Reliability and validity of the Italian version of the 14-item resilience scale. Psychology Research and Behavior Management, 9, 277–284. 10.2147/PRBM.S115657 27757055 PMC5055039

[CIT0010] Camicioli, R., Howieson, D., Lehman, S., & Kaye, J. (1997). Talking while walking. Neurology, 48(4), 955–958. 10.1212/wnl.48.4.955 9109884

[CIT0011] Chertkow, H., Massoud, F., Nasreddine, Z., Belleville, S., Joanette, Y., Bocti, C., Drolet, V., Kirk, J., Freedman, M., & Bergman, H. (2008). Diagnosis and treatment of dementia: 3. Mild cognitive impairment and cognitive impairment without dementia. CMAJ, 178(10), 1273–1285. 10.1503/cmaj.070797 18458258 PMC2335177

[CIT0012] Christensen, K., Doblhammer, G., Rau, R., & Vaupel, J. W. (2009). Ageing populations: The challenges ahead. Lancet (London, England), 374(9696), 1196–1208. 10.1016/S0140-6736(09)61460-4 19801098 PMC2810516

[CIT0013] Cosco, T. D., Kaushal, A., Richards, M., Kuh, D., & Stafford, M. (2016). Resilience measurement in later life: A systematic review and psychometric analysis. Health and Quality of Life Outcomes, 14(1), 1–6. 10.1186/s12955-016-0418-6 26821587 PMC4730639

[CIT0014] Dazzi, C., Pedrabissi, L., & Santinello, M. (2004). Adattamento Italiano delle Scale di Personalità Eysenck per Adulti [Italian Adaptation of the Eysenck Personality Scales for Adults]. Organizzazioni Speciali.

[CIT0015] Devanand, D. P., Kim, M. K., Paykina, N., & Sackeim, H. A. (2002). Adverse life events in elderly patients with major depression or dysthymic disorder and in healthy-control subjects. The American Journal of Geriatric Psychiatry, 10(3), 265–274. 10.1097/00019442-200205000-00005 11994213

[CIT0016] Escourrou, E., Durrieu, F., Chicoulaa, B., Dupouy, J., Oustric, S., Andrieu, S., & Gardette, V. (2020). Cognitive, functional, physical, and nutritional status of the oldest old encountered in primary care: A systematic review. BMC Family Practice, 21(1), 58. 10.1186/S12875-020-01128-7 32220228 PMC7099824

[CIT0017] Farina, F. R., Booi, L., Occhipinti, J. A., Quoidbach, V., Destrebecq, F., Muniz-Terrera, G., & Eyre, H. A. (2023). Young adult brain capital: A new opportunity for dementia prevention. Journal of Alzheimer's Disease, 94(2), 415–423. 10.3233/JAD-230260 37302036

[CIT0018] Fossati, A., Feeney, J. A., Donati, D., Donini, M., Novella, L., Bagnato, M., Acquarini, E., & Maffei, C. (2003). On the dimensionality of the attachment style questionnaire in Italian clinical and nonclinical participants. Journal of Social and Personal Relationships, 20(1), 55–79. 10.1177/02654075030201003

[CIT0019] Fratiglioni, L., Marseglia, A., & Dekhtyar, S. (2020). Ageing without dementia: Can stimulating psychosocial and lifestyle experiences make a difference? The Lancet Neurology, 19(6), 533–543. 10.1016/S1474-4422(20)30039-9 32470425

[CIT0020] Guaita, A., Colombo, M., Vaccaro, R., Fossi, S., Vitali, S. F., Forloni, G., Polito, L., Davin, A., Ferretti, V. V., & Villani, S. (2013). Brain aging and dementia during the transition from late adulthood to old age: Design and methodology of the “Invece.Ab” population-based study. BMC Geriatrics, 13(1), 98. 10.1186/1471-2318-13-98 24063518 PMC3849204

[CIT0021] Hachinski, V., & Avan, A. (2022). From dementia to eumentia: A new approach to dementia prevention. Neuroepidemiology, 56(3), 151–156. 10.1159/000525219 35613542

[CIT0022] Katz, S., Downs, T. D., Cash, H. R., & Grotz, R. C. (1970). Progress in development of the index of ADL. The Gerontologist, 10(1), 20–30. 10.1093/geront/10.1_part_1.20 5420677

[CIT0023] Kivipelto, M., Mangialasche, F., & Ngandu, T. (2018). Lifestyle interventions to prevent cognitive impairment, dementia and Alzheimer disease. Nature Reviews Neurology, 14(11), 653–666. 10.1038/s41582-018-0070-3 30291317

[CIT0024] Laird, K. T., Krause, B., Funes, C., & Lavretsky, H. (2019). Psychobiological factors of resilience and depression in late life. Translational Psychiatry, 9(1), 88. 10.1038/s41398-019-0424-7 30765686 PMC6375932

[CIT0025] Lawton, M. P., & Brody, E. M. (1969). Assessment of older people: Self-maintaining and instrumental activities of daily living. The Gerontologist, 9(3), 179–186. 10.1093/GERONT/9.3_PART_1.179 5349366

[CIT0026] Lemmink, K. A. P. M., Han, K., De Greef, M. H. G., Rispens, P., & Stevens, M. (2001). Reliability of the Groningen fitness test for the elderly. Journal of Aging and Physical Activity, 9(2), 194–212. 10.1123/JAPA.9.2.194

[CIT0027] Lisko, I., Kulmala, J., Annetorp, M., Ngandu, T., Mangialasche, F., & Kivipelto, M. (2021). How can dementia and disability be prevented in older adults: Where are we today and where are we going? Journal of Internal Medicine, 289(6), 807–830. 10.1111/joim.13227 33314384 PMC8248434

[CIT0028] Livingston, G., Huntley, J., Sommerlad, A., Ames, D., Ballard, C., Banerjee, S., Brayne, C., Burns, A., Cohen-Mansfield, J., Cooper, C., Costafreda, S. G., Dias, A., Fox, N., Gitlin, L. N., Howard, R., Kales, H. C., Kivimäki, M., Larson, E. B., Ogunniyi, A., … Mukadam, N. (2020). Dementia prevention, intervention, and care: 2020 report of the Lancet Commission. Lancet (London, England), 396(10248), 413–446. 10.1016/S0140-6736(20)30367-6 32738937 PMC7392084

[CIT0029] MacLeod, S., Musich, S., Hawkins, K., Alsgaard, K., & Wicker, E. R. (2016). The impact of resilience among older adults. Geriatric Nursing (New York, N.Y.), 37(4), 266–272. 10.1016/j.gerinurse.2016.02.014 27055911

[CIT0030] Minnai, F., De Bellis, G., Dragani, T. A., & Colombo, F. (2022). COVID-19 mortality in Italy varies by patient age, sex and pandemic wave. Scientific Reports, 12(1), 1–9. 10.1038/s41598-022-08573-7 35301379 PMC8929285

[CIT0031] Mount, S., Ferrucci, L., Wesselius, A., Zeegers, M. P., & Schols, A. M. W. J. (2019). Measuring successful aging: an exploratory factor analysis of the InCHIANTI Study into different health domains. Aging, 11(10), 3023–3040. 10.18632/AGING.101957 31128067 PMC6555461

[CIT0032] Osborne, J. W. (2014). Best practices in exploratory factor analysis. CreateSpace Independent Publishing.

[CIT0033] Parmelee, P. A., Thuras, P. D., Katz, I. R., & Lawton, M. P. (1995). Validation of the cumulative illness rating scale in a geriatric residential population. Journal of the American Geriatrics Society, 43(2), 130–137. 10.1111/j.1532-5415.1995.tb06377.x 7836636

[CIT0034] Patrizio, E., Calvani, R., Marzetti, E., & Cesari, M. (2021). Physical functional assessment in older adults. The Journal of Frailty & Aging, 10(2), 141–149. 10.14283/jfa.2020.61 33575703

[CIT0035] Petersen, R. C., & Morris, J. C. (2004). Mild cognitive impairment as a clinical entity and treatment target. Archives of Neurology, 62(7), 1160–1163; discussion 1167. 10.1001/archneur.62.7.1160 16009779

[CIT0036] Pietromonaco, P. R., & Beck, L. A. (2019). Adult attachment and physical health why attachment matters for health. Current Opinion in Psychology, 25, 115–120. 10.1016/j.copsyc.2018.04.004 29734091 PMC6191372

[CIT0037] Pinto, J. O., Peixoto, B., Dores, A. R., & Barbosa, F. (2024). Measures of cognitive reserve: An umbrella review. The Clinical Neuropsychologist, 38, 42–115. 10.1080/13854046.2023.2200978 37073431

[CIT0038] Resnick, B., Galik, E., Dorsey, S., Scheve, A., & Gutkin, S. (2011). Reliability and validity testing of the physical resilience measure. The Gerontologist, 51(5), 643–652. 10.1093/geront/gnr016 21402647

[CIT0039] Rolandi, E., Zaccaria, D., Vaccaro, R., Abbondanza, S., Pettinato, L., Davin, A., & Guaita, A. (2020). Estimating the potential for dementia prevention through modifiable risk factors elimination in the real-world setting: A population-based study. Alzheimer’s Research and Therapy, 12(1), 94. 10.1186/s13195-020-00661-y PMC7414752 32767997

[CIT0040] Sachdev, P. S., Blacker, D., Blazer, D. G., Ganguli, M., Jeste, D. V., Paulsen, J. S., & Petersen, R. C. (2014). Classifying neurocognitive disorders: The DSM-5 approach. Nature Reviews Neurology, 10(11), 634–642. 10.1038/nrneurol.2014.181 25266297

[CIT0041] Schröder, H., Fitó, M., Estruch, R., Martínez‐González, M. A., Corella, D., Salas‐Salvadó, J., Lamuela‐Raventós, R., Ros, E., Salaverría, I., Fiol, M., Lapetra, J., Vinyoles, E., Gómez‐Gracia, E., Lahoz, C., Serra‐Majem, L., Pintó, X., Ruiz‐Gutierrez, V., & Covas, M. (2011). A short screener is valid for assessing Mediterranean diet adherence among older Spanish men and women. The Journal of Nutrition, 141(6), 1140–1145. 10.3945/jn.110.135566 21508208

[CIT0042] Song, S., Stern, Y., & Gu, Y. (2022). Modifiable lifestyle factors and cognitive reserve: A systematic review of current evidence. Ageing Research Reviews, 74, 101551. 10.1016/j.arr.2021.101551 34952208 PMC8794051

[CIT0043] Stern, Y. (2012). Cognitive reserve in ageing and Alzheimer’s disease. The Lancet Neurology, 11(11), 1006–1012. 10.1016/S1474-4422(12)70191-6 23079557 PMC3507991

[CIT0044] Vaccaro, R., Borrelli, P., Abbondanza, S., Davin, A., Polito, L., Colombo, M., Francesca Vitali, S., Villani, S., & Guaita, A. (2017). Subthreshold depression and clinically significant depression in an Italian population of 70–74-year-olds: Prevalence and association with perceptions of self. Biomed Research International, 2017, 1–8. 10.1155/2017/3592359 PMC5368375 28393076

[CIT0045] Vaupel, J. W. (2010). Biodemography of human ageing. Nature, 464(7288), 536–542. 10.1038/nature08984 20336136 PMC4010874

[CIT0046] Viña, J., Borrás, C., & Miquel, J. (2007). Theories of ageing. IUBMB Life, 59(4–5), 249–254. 10.1080/15216540601178067 17505961

[CIT0047] Walsh, E., Blake, Y., Donati, A., Stoop, R., & Von Gunten, A. (2019). Early secure attachment as a protective factor against later cognitive decline and dementia. Frontiers in Aging Neuroscience, 11(JUL), 1–20. 10.3389/fnagi.2019.00161 31333443 PMC6622219

[CIT0048] Whitson, H. E., Duan-Porter, W., Schmader, K. E., Morey, M. C., Cohen, H. J., & Colón-Emeric, C. S. (2016). Physical resilience in older adults: Systematic review and development of an emerging construct. The Journals of Gerontology. Series A, Biological Sciences and Medical Sciences, 71(4), 489–495. 10.1093/gerona/glv202 26718984 PMC5014191

[CIT0049] Ye, K. X., Sun, L., Wang, L., Khoo, A. L. Y., Lim, K. X., Lu, G., Yu, L., Li, C., Maier, A. B., & Feng, L. (2023). The role of lifestyle factors in cognitive health and dementia in oldest-old: A systematic review. Neuroscience and Biobehavioral Reviews, 152, 105286. 10.1016/j.neubiorev.2023.105286 37321363

