# Supplementary material for: Lifestyle, Cognitive, and Psychological Factors Associated With a Resilience Phenotype in Aging: A Multidimensional Approach on a Population-Based Sample of Oldest-Old (80+)
Source: J Gerontol B Psychol Sci Soc Sci. 2024 Aug 3;79(10):gbae132. doi: 10.1093/geronb/gbae132 (PMC11402365; doi:10.1093/geronb/gbae132)
Supplement: gbae132_suppl_Supplementary_Materials [file gbae132_suppl_supplementary_materials.docx]

***The Journals of Gerontology, Series B: Psychological Sciences and Social Sciences* Supplementary Material: Rolandi et al. Lifestyle, cognitive, and psychological factors associated with a resilience phenotype in aging: a multidimensional approach on a population-based sample of oldest-old (80+).**

**Supplementary Table 1**: Factor Correlation matrix

| **Factor** | **Cognitive reserve** | **Affective reserve** | **Insecure attachment** | **Current lifestyle** | **Physical reserve** | **Avoidant attachment** |
| --- | --- | --- | --- | --- | --- | --- |
| Cognitive reserve | - | -.017 | -.201 | -.216 | -.003 | -.094 |
| Affective reserve |  | - | .099 | -.186 | .183 | .025 |
| Insecure attachment |  |  | - | .156 | -.015 | .048 |
| Current lifestyle |  |  |  | - | -.149 | .144 |
| Physical reserve |  |  |  |  | - | -.180 |
| Avoidant attachment |  |  |  |  |  | - |
